# Evolutionary Trajectories of Complex Traits in European Populations of Modern Humans

**DOI:** 10.3389/fgene.2022.833190

**Published:** 2022-03-28

**Authors:** Yunus Kuijpers, Jorge Domínguez-Andrés, Olivier B. Bakker, Manoj Kumar Gupta, Martin Grasshoff, Cheng-Jian Xu, Leo A.B. Joosten, Jaume Bertranpetit, Mihai G. Netea, Yang Li

**Affiliations:** ^1^ Centre for Individualised Infection Medicine, CiiM, A Joint Venture Between the Hannover Medical School and the Helmholtz Centre for Infection Research, Hannover, Germany; ^2^ TWINCORE, Centre for Experimental and Clinical Infection Research, A Joint Venture Between the Hannover Medical School and the Helmholtz Centre for Infection Research, Hannover, Germany; ^3^ Department of Internal Medicine and Radboud Center for Infectious Diseases (RCI), Radboud University Nijmegen Medical Centre, Nijmegen, Netherlands; ^4^ Radboud Institute for Molecular Life Sciences (RIMLS), Radboud University Medical Center, Nijmegen, Netherlands; ^5^ Department of Genetics, University Medical Centre Groningen, Nijmegen, Netherlands; ^6^ Institut de Biologia Evolutiva (UPF-CSIC), Universitat Pompeu Fabra, Barcelona, Spain; ^7^ Department for Genomics and Immunoregulation, Life and Medical Sciences Institute (LIMES), University of Bonn, Bonn, Germany

**Keywords:** evolution, complex traits, Neolithic revolution, human genetics, polygenic risk scores (PRS)

## Abstract

Humans have a great diversity in phenotypes, influenced by genetic, environmental, nutritional, cultural, and social factors. Understanding the historical trends of physiological traits can shed light on human physiology, as well as elucidate the factors that influence human diseases. Here we built genome-wide polygenic scores for heritable traits, including height, body mass index, lipoprotein concentrations, cardiovascular disease, and intelligence, using summary statistics of genome-wide association studies in Europeans. Subsequently, we applied these scores to the genomes of ancient European populations. Our results revealed that after the Neolithic, European populations experienced an increase in height and intelligence scores, decreased their skin pigmentation, while the risk for coronary artery disease increased through a genetic trajectory favoring low HDL concentrations. These results are a reflection of the continuous evolutionary processes in humans and highlight the impact that the Neolithic revolution had on our lifestyle and health.

## Introduction

Important human traits such as height, body mass index (BMI), or diseases susceptibility vary greatly between populations, both geographically and temporally. Local conditions on different continents and various climates influence human phenotypes through a combination of environmental and genetic factors (Przeworski, 2002; Byars et al., 2010). Understanding how local conditions shape contemporary humans’ genome and phenotype will help us to predict future modifications for specific traits having medical significance and we can also make short-term predictions about our future evolution and adaptation (Byars et al., 2010). This is important not only for understanding contemporary human’s physiology but also for adapting public health measures that are tailored for specific populations (Sabeti et al., 2002; Byars et al., 2010). For example, Asian populations present an increased risk of metabolic and cardiovascular complications at a lower BMI compared to European populations (Rao et al., 2015), which has important consequences for the implementation of proper public health prevention strategies. Moreover, the massive rural-to-urban transition now taking place in the developing world needs to be understood at the level of changes in physiological phenotypes and subsequently accompanied by measures to prevent diseases of modern societies (Neiderud, 2015). In this respect, understanding historical trends of important human physiological traits will help to decipher human physiology in general, and to disentangle the factors that influence human diseases in modern societies in particular (Sabeti et al., 2002, Sabeti et al., 2006; Tang et al., 2007).

A very limited number of complex traits, most notably height, can be relatively easily assessed on historical samples based on physical measurements of skeletal remains in different populations during history. Such studies have shown that the height of modern humans decreased from the Early Upper Paleolithic to Mesolithic period and thereafter slightly increased towards the Bronze Age (Rosenstock et al., 2019). Interestingly, this observation has been validated using “genetic height scores” using information from genome-wide association studies (GWAS) in large cohorts of modern human populations (Cox et al., 2019). Few other heritable traits have also been assessed in ancient individuals using the assessment of gene alleles associated with particular phenotypes, most often skin pigmentation and eye color (Olalde et al., 2014; Field et al., 2016). In 2014,Olalde et al. (2014) sequenced a ∼7,000 year-old Mesolithic skeleton discovered at the La Braña-Arintero site in León, Spain, to retrieve a complete genome of pre-agricultural European humans. Analyses including Outgroup f3 (Reich et al., 2009), PCA (Patterson et al., 2006), and D statistics (Green et al., 2010) were carried out to understand the relationship between this genome and other ancient samples. In another study, Field and the team (Field et al., 2016) introduced the singleton density score (SDS) to detect recent allele frequency modifications at the single-nucleotide polymorphisms level in contemporary human populations. These studies clearly demonstrate the power of genetic information to predict the trajectories of important human traits during history, although no systematic studies have been performed yet.

GWAS has also identified thousands of genomic variants that are significantly associated with diverse phenotypes (Tam et al., 2019). Although the individual effect of each of these variants is limited, the combined effect of multiple associated variants (so-called polygenic risk scores, PRS) can be used as a powerful tool for the prediction of certain traits (de Jong et al., 2018; Cox et al., 2019). For example, the combination of all the height-associated variants can explain up to 45% of the total variance in a European population (Yang et al., 2010). PRS consists of the summed effects of all independent genetic variants associated with a specific trait (e.g., height), and it can be therefore regarded as a representation of a specific predicted phenotype based on DNA (Torkamani et al., 2018). Of note, the predictive power of PRS increases with the similarity of the populations in which GWAS was performed (Cox et al., 2019). Considering this, in the present study, we estimated PRS in ancient European populations for a series of heritable traits, including height, BMI, lipoprotein concentrations, cardiovascular disease, and intelligence, using large summary statistic databases of GWAS published in the literature. We aimed to investigate the PRS trajectiory in European populations of modern *Homo sapiens* since the Early Upper Paleolithic, through Mesolithic, Neolithic, Bronze Age, medieval, and modern time periods.

## Materials and Methods

### Cohort Selection

Ancient DNA genotype data was downloaded from version 37.2 of the published aDNA genotype database, compiled by and available on the David Reich Lab website. The ancient DNA samples consisted of pseudo-haploid genotype data. This was due to the low genotyping coverage. Samples with variant missingness above 96 percent were filtered out using Plink version 1.9 (Purcell et al., 2007). This was done in order to remove outliers with extremely low coverage. Only samples within Europe were used for this study; these samples were selected based on their geographic location, which is latitude (within 35 and 70 degrees north) and longitude (within 10 degrees west and 40 degrees east). Samples without a carbon-dated age were also filtered out. We also selected 250 European samples from the 1,000 genomes to project phase 3. Only variants present in both the ancient samples and the modern samples were retained. This resulted in a dataset of 827 ancient samples and 250 modern samples containing 1,233,013 variants. The total number of filtered SNPs for each trait shown in our main findings is provided in Supplementary Table S3 (column N).

### Carbon-Dated Sample Origin and Geographical Location

Both carbon-dated age of origin, as well as latitudinal and longitudinal data, was available for these 827 ancient European samples. Broad time periods were assigned to these samples with the Early Upper Paleolithic era for all samples originating from before 25,000 years before the common era (BCE) standardized to 1950. The Late Upper Paleolithic era follows until 11,000 BCE. The Mesolithic era ranges from 11,000 to 5500 BCE. The Neolithic era ranges from 8,500 to 3900 BCE, and the Post-Neolithic era ranges from 5000 BCE and more recent ages. Using the geographical data in combination with archeological clues and the genetic data, the broad time period of origin was also available for samples that were dated to a point in time with overlapping broad time periods. This allowed the samples to be classified as either Early Upper Paleolithic, Late Upper Paleolithic, Mesolithic, Neolithic, or Post-Neolithic. The sample age of the 250 modern European samples was set to 0.

### Summary Statistic Selection

Summary statistics for complex traits, like, standing height, skin tanning ease, skin color, BMI, and intelligence, were obtained from the United Kingdom Biobank and the GWAS catalog. The intelligence score of each participant was assessed employing different neurocognitive tests, SAT test scores, and WAIS IQ score (Savage et al., 2018). Some complex traits had multiple different sets of summary statistics available. In these cases, the data which was more recent and used bigger cohorts that were either of European or mixed (European and Asian) ancestry were selected. The variants of these summary statistics were then filtered by only keeping bi-allelic variants. Most aDNA genotypes available are pseudo-haploid as a consequence of their lower sample quality. We excluded ambiguous SNPs (A/T and C/G) in order to prevent errors due to strand flips present in these pseudo-haploid samples.

### Polygenic Risk Scores Calculation

Polygenic risk scores were then calculated by first intersecting the filtered variants from the summary statistics with the variants present in the DNA samples. Starting at the most significant variant, all variants within 250 kb of that variant that was less significant were excluded. We then multiplied the dosage of the remaining variants with the effect size and these values were summed. If a variant is missing in a sample, the dosage is substituted with the average genotyped dosage for that variant within the entire dataset. This way, the PRS is not skewed in any specific direction. The formula for this is described below with the score *S* being the weighted sum of a variant’s dosage *X*_*n*_ multiplied by its associated weight or beta *β*_*n*_ calculated using *m* variants.

$$S=\sum_{n=1}^{m}X_{n}\;\beta_{n}$$

### Relation Between Polygenic Risk Scores and Carbon-Dated Sample Age

We constructed piecewise linear models for each trait by separating the samples into two groups. These two groups consisted of all samples preceding the Neolithic era and those of the Neolithic era and later, respectively. We correlated PRS with the carbon-dated age of our samples. We then multiplied the -log10 of the Pearson correlation *p* values with the sign of the correlation coefficients.

In addition, we plotted LOESS regression models to highlight the change in PRS at each point in time independent of any predefined breakpoint between historical periods. We also performed a group-based comparison using a student *t*-test. We compared pre-Neolithic, Neolithic, post-Neolithic, and Mesolithic samples with their respective adjacent historical periods to show the difference between other historical transitions besides the pre- and post-Neolithic.

### Selective Pressure Test

We tested whether traits observed changes were due to selective pressure during adjacent time periods. We performed a two-tailed test using the mean F_st_ calculated with trait-specific SNP’s between two adjacent periods and a reference distribution of 10,000 random LD and MAF matched mean F_st_ scores calculated using an equal amount of SNP’s (Wright, 1951). Bonferroni correction was performed to account for multiple testing.

### Robustness of Results

In order to test the robustness of our results, we calculated PRS using multiple different *p*-value thresholds for QTL inclusion. We used *p*-value thresholds from 10^–5^ to 10^–8^ for the complex traits obtained through the GWAS catalog and the United Kingdom Biobank. We also calculated PRS using different variant missingness thresholds. This means we removed samples with a variant missingness rate higher than 96, 90, 80, or 70 percent. All of the results from the piecewise linear models were then used to create a heatmap depicting the consistency and robustness of our observed correlations.

Additionally, various window sizes were used for clumping the QTL’s and LD-based clumping was also performed, excluding variants with an LD greater than 0.2 compared to our lead SNP within a window. In order to see whether our observations were due to sample imbalances between the pre-Neolithic period and the later periods, samples originating from the Neolithic period and later were randomly down-sampled to the same number of samples as the pre-Neolithic samples. We then recalculated the correlation coefficients and compared the significance and the direction of the correlation between the down-sampled set and the full dataset.

## Results

**FIGURE 1:**
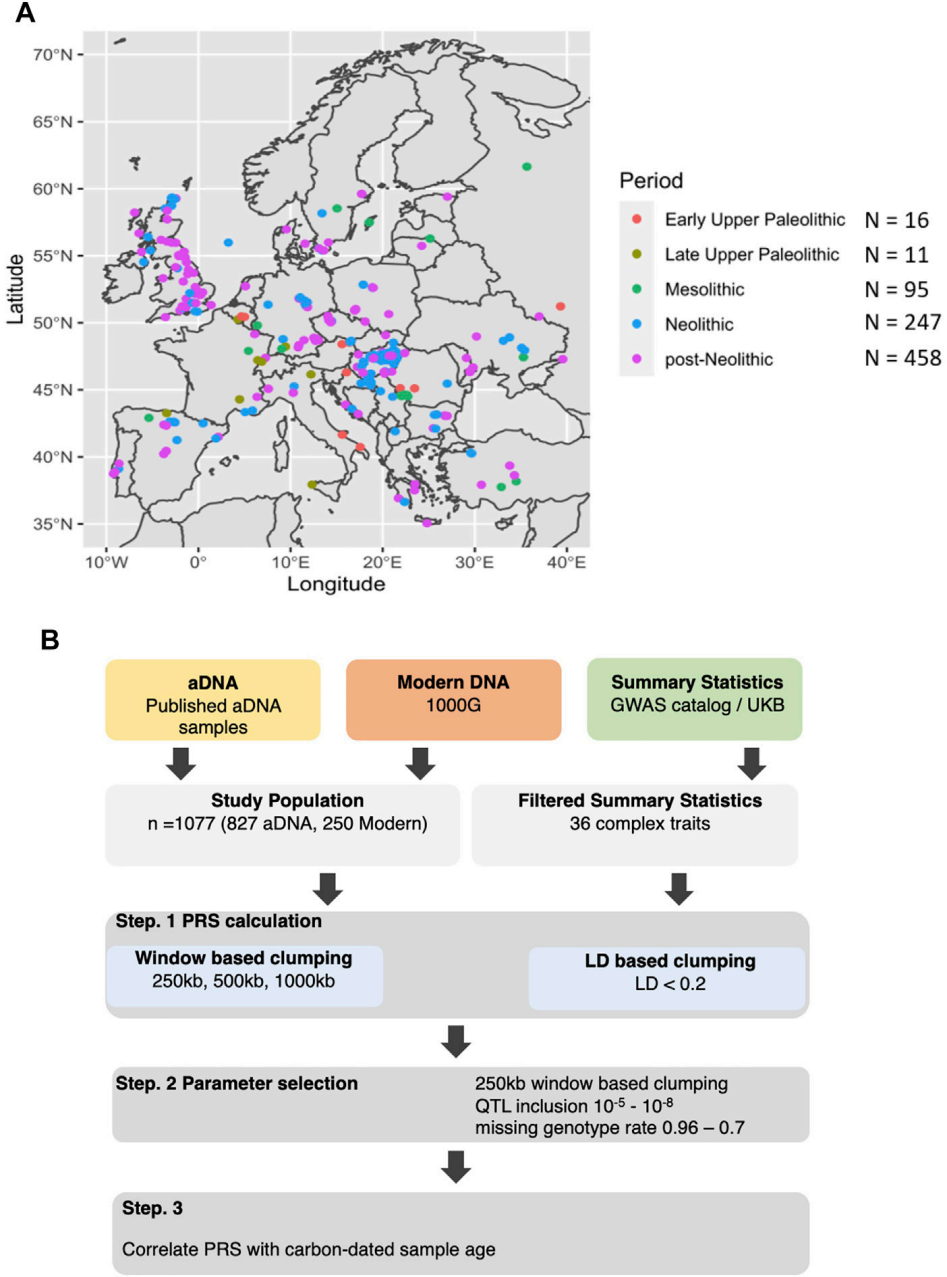
Origin of the samples and analysis performed. **(A)** Distribution of the ancient DNA samples across Europe colored by a broad historical period ranging from Early Upper Paleolithic, Late Upper Paleolithic, Mesolithic, Neolithic, and post-Neolithic. **(B)** Both aDNA and modern DNA samples of European individuals were used in combination with summary statistics from predominantly European populations to calculate PRS. This was done at various threshold combinations before correlating the scores with the sample age.

We aimed to investigate the PRS trajectory for a series of heritable (Supplementary Table S1) in European populations of modern *Homo sapiens* since the Early Upper Paleolithic, through Mesolithic, Neolithic, Bronze Age, medieval, and modern time periods. The different origins of the samples allowed us to cover the majority of the territory of Europe, including areas of Scandinavia, the United Kingdom, Central and Eastern Europe, the Mediterranean area, and Anatolia (Figure 1A). In order to increase the matching of our populations, we employed summary statistics exclusively derived from European cohorts and European aDNA samples, which in turn increased the predictive power of our results. However, it is important to note that the absolute values of the PRS will not be accurate enough to make any direct conclusions due to the limited heritability of many complex traits, for instance, the heritability of height is 79% and BMI:40% (Geddes, 2019) (Supplementary Table S2). Therefore, we focused on the direction of the changes observed in the PRS within the entire dataset to model the changes in the genetic regulation of these traits within these specific populations.

We used ancient DNA (aDNA) data of 827 ancient individuals from Western Eurasia, which were publicly available and aggregated into a database released 22 February 2019, by the David Reich lab (version 37.2) (https://reich.hms.harvard.edu/allen-ancient-dna-resource-aadr-downloadable-genotypes-present-day-and-ancient-dna-data). The aDNA data was used in conjunction with DNA data of 250 randomly selected modern individuals from Western Europe, which were downloaded from 1000G database (Auton et al., 2015),. Using GWAS summary statistics from GWAS catalogue (MacArthur et al., 2017) and United Kingdom Biobank (https://www.nealelab.is/uk-biobank), which can be seen in Supplementary Table S1, we calculated PRS for each individual for various traits respectively, using a QTL *p*-value threshold of 10^–6^, in 250 kb windows. Multiple thresholds were further tested for assuring the robustness of the findings. We supplemented the effect of missing variants in a sample with the average of the entire population. We then scaled the PRS distribution to have a range of 1 to −1 before correlating them with the carbon-dated sample age. The general overview of the analysis can be seen in Figure 1B.

One general observation is the existence of a clear difference in the trajectories of the various traits before and after the Neolithic revolution: few changes are seen between the Early Upper Paleolithic until the Neolithic period, with a general acceleration of the evolutionary processes thereafter. But, a group-based comparison between adjacent time periods shows significant changes between the pre-Neolithic and the Neolithic samples for several traits. However, this approach loses the information regarding the carbon-dated age of each individual sample (e.g., the Paleolithic-Mesolithic grouping spans approximately 40,000 years in Europe, and trends in this period are lost). A down-sampling analysis shows that despite the reduction in power, due to the lower sample size in linear regression models, the correlation coefficients remain significantly similar between the complete dataset and the down-sampled dataset (Supplementary Figure S1). Lastly, in order to validate the robustness of our results, all PRS models were constructed using multiple different threshold combinations for both the missing genotype rate of the samples, the GWAS *p*-value threshold, as well as the window-size of each independent region. These results show our observations are consistent across multiple thresholds (Supplementary Figure S2).

**FIGURE 2:**
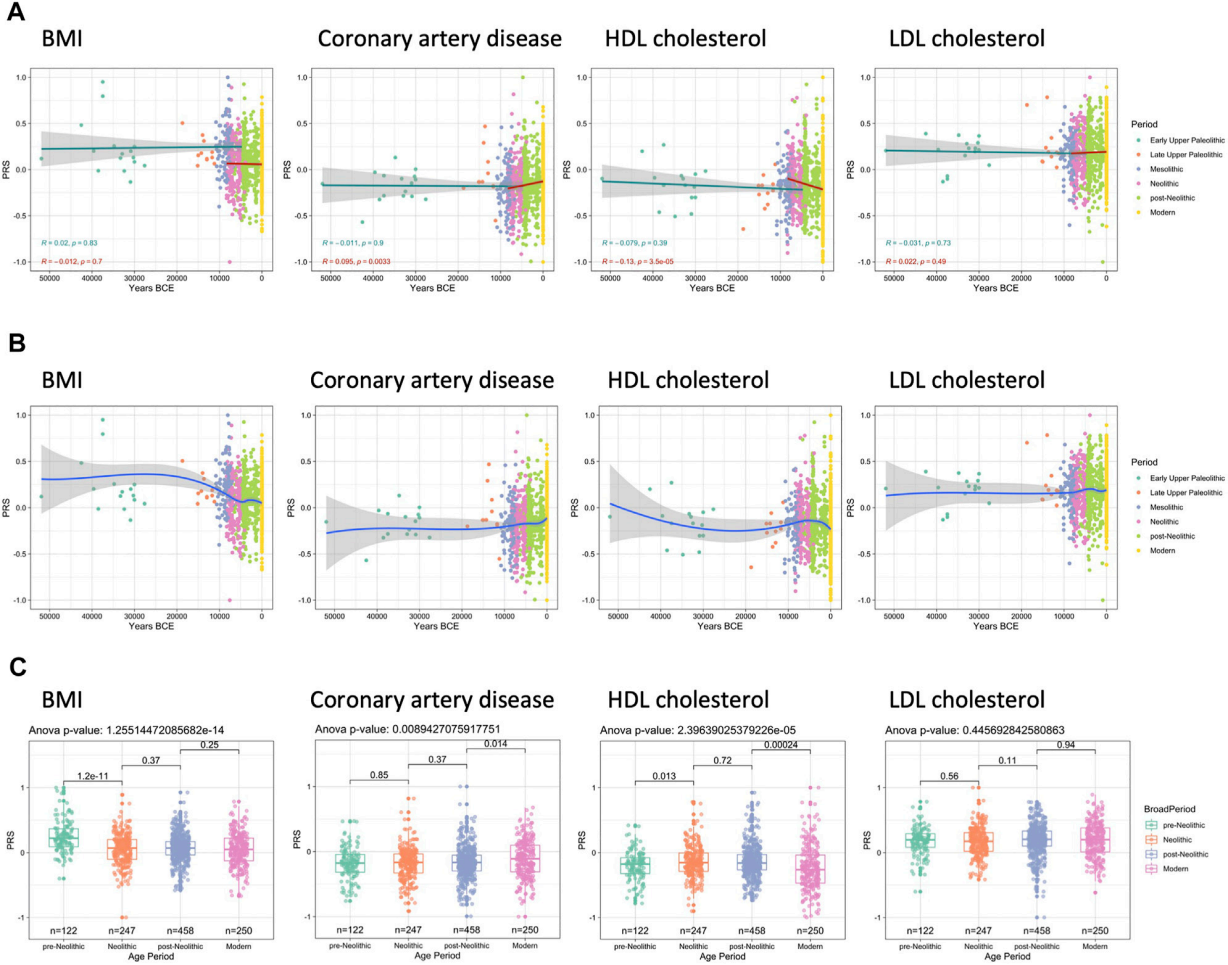
Trajectories of PRS linked with body mass index (BMI), coronary artery disease, and cholesterol. Max missing genotype per sample: 0.96. QTL *p* value cutoff: 10^–6^. **(A)** Prior to the Neolithic revolution no significant changes in PRS are observed; however, after the start of the Neolithic period a significant increase in coronary artery disease PRS can be seen as well as a decrease in HDL cholesterol PRS. **(B)** LOESS regression models show that BMI PRS mostly changes during the Mesolithic and Neolithic, whereas the decrease in HDL cholesterol PRS was mostly occurring in the post-Neolithic and modern era. **(C)** These differences are also significant when comparing the different broad periods as a whole.

The first set of traits in which this pattern is apparent are height and skin color. The genetic modeling shows that standing height remained relatively constant between the Paleolithic and Neolithic periods (Supplementary Figure S4). The second set of analyses was focused on skin color and skin tanning ease. In accordance with earlier studies (Olalde et al., 2014), we have also detected a surprising maintenance of dark skin through Paleolithic and Mesolithic (Supplementary Figure S4). In the last few centuries, Europe has experienced a significant rural-to-urban transition, which in turn have also affected their diet and lifestyle and made them prone to metabolic and cardiovascular diseases (Anand et al., 2015). Therefore, we next aimed to model the changes in genetic traits that influence either body weight and BMI or lipid metabolism and cardiovascular complications. The analysis of the trajectory of genetic predisposition to changes in BMI, total cholesterol, high-density lipoprotein (HDL), and low-density lipoprotein (LDL) did not show significant trends before the Neolithic (Figure 2A). However, a comparison between all pre-Neolithic and Neolithic samples grouped together did show a significant decrease in PRS between the time periods (Figure 2C). Interestingly, the evolution of the metabolic pathways associated with the BMI and LDL-cholesterol did not show any changes after the Neolithic either. Interestingly, we observe an increase in the genetic factors that lead to the development of coronary artery disease, which is related to a constant decrease in HDL cholesterol in European populations after the Neolithic revolution (Ali et al., 2012).

**FIGURE 3:**
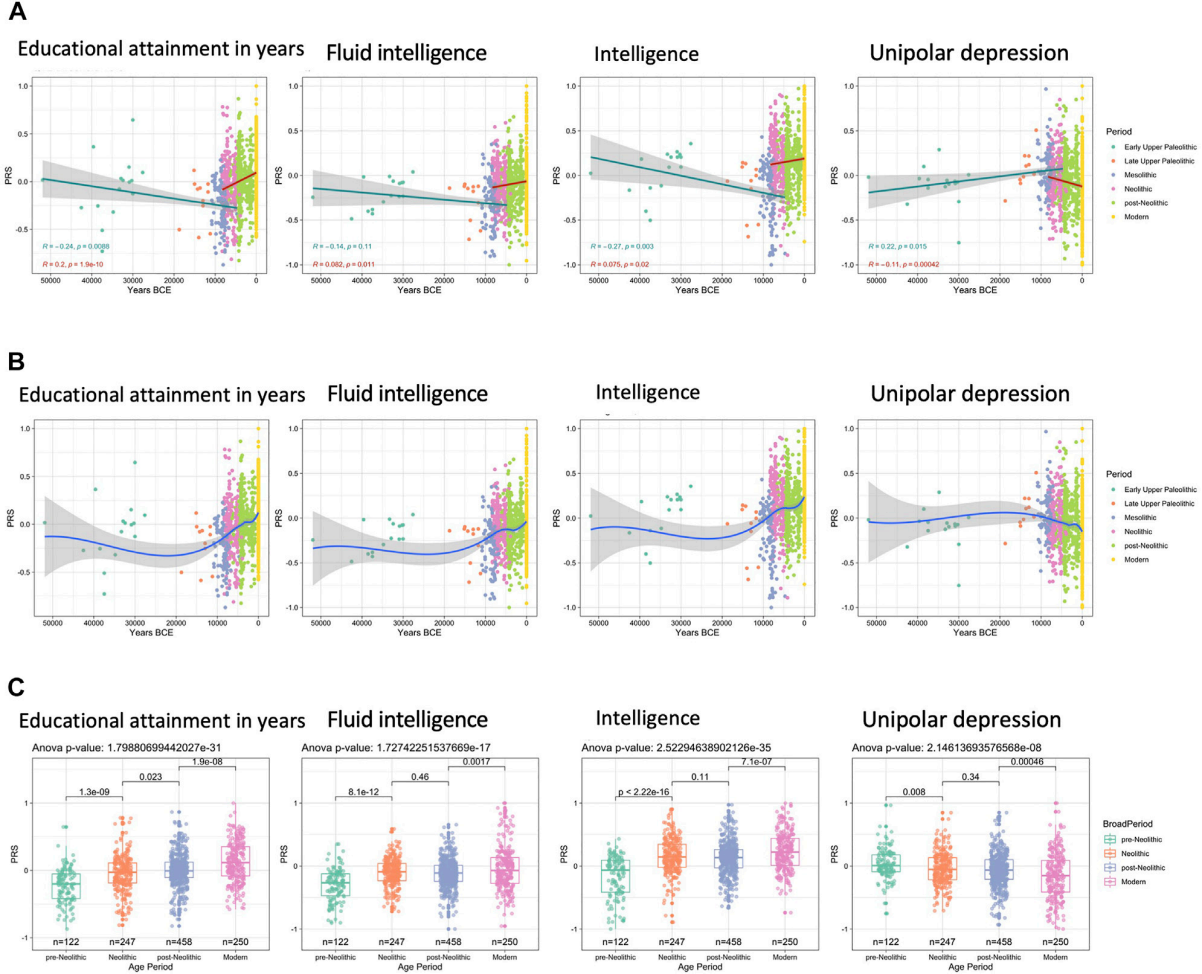
Trajectories of human cognition-related traits. Max missing genotype per sample: 0.96. QTL *p*-value cutoff: 10^–6^. **(A)** Significant decreases in PRS can be seen prior to the Neolithic revolution for all cognition-related traits except fluid intelligence, followed by significant increases in PRS over time. This pattern for cognition-related traits is reversed for unipolar depression. **(B)** LOESS regression models show that between the Mesolithic and Neolithic, and the post-Neolithic and modern era large changes in PRS can be seen, whereas the difference between Neolithic and post-Neolithic is much smaller. **(C)** *t*-tests also show that the changes observed are significantly different to a much larger extent between the pre-Neolithic and Neolithic and between the post-Neolithic and Modern era.

Since, some genetic polymorphisms in cholesterol-related pathways are connected to cognitive functions, while variations in the levels of HDL and LDL have been linked with alterations in intelligence, learning, and memory (Muldoon et al., 1997; Schreurs, 2010), in the last set of analyses, we mainly focused on the evolution of genetic factors related to cognitive functions. While keeping in mind that GWAS studies aiming to characterize a trait as complex as human intelligence are confounded for measuring performance based on the western education system, both the GWAS population, as well as the ancient DNA samples, are of European origin. Interestingly, while the period between the Early Upper Paleolithic and the Neolithic is characterized by stagnation or slight decrease in PRS related to intelligence, the genetic data show a clear increase in the scores for educational attainment, intelligence, and fluid intelligence from the Neolithic onwards, while the traits related with unipolar depression tend to decrease from that era on (Figures 3A,B). The most significant differences can be observed comparing the pre-Neolithic and Neolithic groups, as well as the post-Neolithic and modern groups, whereas the period between the Neolithic and post-Neolithic shows a very constant distribution of PRS scores (Figure 3C).

Finally, as an indication of selection, we used Wright’s fixation index (F_st_) to calculate mean F_st_ scores per trait to analyze if they differed significantly from the distribution of 10,000 LD and MAF matched mean F_st_ scores (Wright, 1951). This was done separately for the period between pre-Neolithic and Neolithic samples, Neolithic and post-Neolithic samples, and post-Neolithic and modern samples (Supplementary Table S3). Traits like educational attainment in years, intelligence, BMI, HDL, LDL, and skin tanning ease show significant selective pressure between pre-Neolithic and Neolithic samples. During the Neolithic to post-Neolithic period, HDL still shows strong selective pressure in contrast to the other metabolic traits. Lastly, between the post-Neolithic and modern samples, BMI, educational attainment in years, fluid intelligence, skin color, skin tanning ease, and standing height show significant levels of selection. It remains important, however, to realize that differences between post-Neolithic and modern samples could in part be explained by differences in sequencing quality which are lower for the fragmented aDNA samples.

## Discussion

Owing to the recent development of high throughput technologies, a plethora of genetic information has been generated for both contemporary human populations as well as ancient DNA samples. This in turn aids us to reconstruct and understand the human’s genetic history more comprehensively, including, “out-of-Africa” expansion and admixture with ancient hominins (Quintana-Murci, 2016). These studies also help us to reveal the degree to which selection acts on the human genome of the different locations, thereby providing detailed insight into how selection removes deleterious variation as well as the capability of human populations to adapt to the wide range of nutritional, climatic, and pathogenic environments they have occupied (Quintana-Murci, 2016). Considering this, in the present study, we aimed to investigate the trajectory of PRS in European human populations across different historical ages. Result obtained reveals that few changes are seen between the Early Upper Paleolithic until the Neolithic period, with a general acceleration of the evolutionary processes thereafter. One possible explanation is methodological, as smaller numbers of genomes were available for the analyses of the early periods of human occupation of Europe, while more genomes (and thus statistical power) are available for the later historical period. However, that is unlikely to be the entire explanation for this difference, as strong changes are seen in immune traits both before and after the Neolithic (Domínguez-Andrés et al., 2021), which demonstrates that the data available are enough to detect clear differences when present. However, a group-based comparison between adjacent time periods shows significant changes between the pre-Neolithic and the Neolithic samples for several traits.

Further, genetic modeling shows that standing height remained relatively constant between the Paleolithic and Neolithic periods (Supplementary Figure S4). This suggests that the decrease in height based on skeletal measurements was strongly driven by environmental factors such as infections and diet. Indeed, it is well known that the increase in population density during the Neolithic revolution led both to a relative worsening of the dietary intake per individual compared to hunter-gatherers, as well as with a significant increase in the infectious diseases burden (Page et al., 2016). In addition, the domestication of animals led to a much larger number of zoonotic diseases, with up to 80% of modern infections being hypothesized to be traced to domesticated animals (Diamond, 2010). Poor caloric intake and a high number of childhood infections are both known to lead to stunting (low height) (Vonaesch et al., 2017). While the beginning of the Neolithic revolution marks the lowest standing height of humans for the reasons detailed above, height increases significantly during the following historical periods (Supplementary Figure S4), a trend that is clearly documented in modern populations as well (Stulp and Barrett, 2016). In 2019, Cox and the team also hypothesized that both skeletal and genetic height remained constant between the Mesolithic and Neolithic and increased between the Neolithic and Bronze Age (Cox et al., 2019). These findings also support the concept of “selection on correlated traits”, which means that when characters are genetically correlated, selection completely on one will reflect in a correlated modification in the second (Falconer and Mackay, 1996).

Subsequent analyses were focused on skin color and skin tanning ease. Earlier studies reported a surprising maintenance of dark skin through Paleolithic and Mesolithic (Olalde et al., 2014), which is also confirmed by our study (Supplementary Figure S4). During the Paleolithic and Mesolithic, there were few changes in skin pigmentation in European hunter-gatherer populations, with Mesolithic Europeans might be retaining dark skin up to 7,000 years ago (Olalde et al., 2014), 30,000 years after the modern *Homo sapiens* migration to Europe (Hoffecker, 2009). Populations with lighter skin might have arrived in Europe with the Neolithic migration from the Middle East, around 8,000 years ago (Mathieson et al., 2018), after which we document a strong decrease in the pigmentation of the skin. Recently, Ju and Mathieson also observed that the dark pigmentation alleles in the European population decreased significantly over the past 40,000 years (Ju and Mathieson, 2021). However, the dark skin pigmentation within the Early Upper Paleolithic European populations might be due to the relatively slow adaptation to the high-latitude conditions arising from the recent migration from lower latitudes.

In parallel with the lighter skin, we observe an increase in the score for the easiness for tanning after the Neolithic revolution. The biological processes behind these changes may be related to vitamin D deficiencies associated with the colder climates of Europe, driving a lighter skin color that improves local production of vitamin D in the skin (Yuen and Jablonski, 2010). However, excessively light skin can cause dermatological problems and loss of folic acid, with deleterious consequences (Jones et al., 2018). In this sense, the increase in skin tanning ease can compensate for the loss of skin pigmentation and protect current European populations from the harmful effects of excessive solar radiation during summer (Branda and Eaton, 1978).

Since the Neolithic revolution also led to significant changes in the diet as a consequence of the development of agriculture and animal domestication. Later on, the rural-to-urban transition taking place in Europe in the last few centuries also led to very significant changes in diet and lifestyle, which subsequently resulted in important changes in the risk of developing metabolic and cardiovascular diseases (Anand et al., 2015). Therefore, further, we model the changes within genetic traits that influence either body weight and BMI or lipid metabolism and cardiovascular complications. Results obtained did not show significant trends before the Neolithic (Figure 2A).

Further comparison between all pre-Neolithic and Neolithic samples grouped together the evolution of the metabolic pathways associated with the BMI and LDL-cholesterol did not show any changes after the Neolithic. Overall, these results argue against the ‘thrifty gene hypothesis,’ which states that natural selection has driven the fixation of genetic variants that favor a thrifty metabolism (Hales and Barker, 2001). Our results are, therefore, in agreement with previously published reports which analyzed the positive selection of SNPs associated with BMI and did not support the idea that a thrifty metabolism provided an evolutionary advantage (Wang and Speakman, 2016).

Interestingly, we also observe an increase in the genetic factors that lead to the development of coronary artery disease, which is related to a constant decrease in HDL cholesterol in European populations after the Neolithic revolution (Ali et al., 2012). If this adaptation causes disease, we could wonder why it might be evolutionarily advantageous to have lower HDL cholesterol concentrations. A reason for this could be related to cognitive functions since cholesterol is fundamental for the development and functioning of the brain. Recent study also reported that, for LDL, the trait increasing allele is more common in European populations (Mathieson and Mathieson, 2018). Some genetic polymorphisms in cholesterol-related pathways are connected to cognitive functions, while variations in the levels of HDL and LDL have been linked with alterations in intelligence, learning, and memory, although the full implications and the mechanisms are still far from being understood (Muldoon et al., 1997; Schreurs, 2010).

Since, some genetic polymorphisms in cholesterol-related pathways are connected to cognitive functions, in the last set of analyses, the evolution of genetic factors related to cognitive functions were estimated. Result obtained reveals that increased cognitive functions are an evolutionary advantage to adapt to the environment. The strong increase in social complexity resulting from the Neolithic revolution and the process of urbanization and occupational specialization are likely factors that could have driven the evolutionary advantage of improved intelligence-related scores. The decrease in the score for unipolar depression is likely a mirror of that process as well, as it is known that depression is associated with a lower IQ score (Melby et al., 2020). As for the changes in PRS for the cognitive traits we included in this study it is important to put these in perspective. The measure of educational attainment in years is largely a trait influenced by socioeconomic factors so the changes we observe only affect approximately 20% of the actual variation we observe in this trait as reported in the original GWAS by Okbay et al. (2016). For the fluid intelligence test performed in the UK-Biobank a similar heritability is estimated although the test itself only consists of 13 questions severely limiting the reliability of this specific test. The overall Intelligence reported by Savage et al. (2018) refers to a meta-analysis of various different tests that aim to capture overall intelligence and though the heritability is reported to be approximately 60% for intelligence this trait might be less accurate due to the heterogeneity of the tests included in the meta-analysis. Similarly, the measure of unipolar depression is also based on a meta-analysis by Nagel et al. (2018) that compromised various measures from the UK-Biobank the Genetics of Personality Consortium collected through the use of various questionnaires which means the reliability of this trait as measure of depression is hard to ascertain.

In short, although we see an increase in PRS for cognitive functions over time this does not necessarily translate to an evolutionary pressure towards an increasing intelligence. What this means is that there is an increase in allelic frequencies for alleles that positively impact multiple different measures of cognition but only to a limited extent in relation with the heritability of these traits.”

The evolutionary processes that have led to an increase in intelligence during the last 10,000 years are likely different from the recent gains in cognitive performance observed in the last century, including intelligence, episodic memory, and semantic memory (Rönnlund and Nilsson, 2009): the so-called Flynn effects. Since IQ began to be used at the beginning of the 20th century to assess human intelligence, every year the IQ scores are higher, with an average increase of 2.8 points per decade (Trahan et al., 2014). While the Flynn effect is most likely explained by environmental (and possible epigenetic) factors, the genetic-based evolutionary pressure on intelligence in humans is thus confirmed in modern humans since the advent of agriculture and the process of urbanization. Finally, Wright’s fixation index (Fst) analysis reveals that HDL shows strong selective pressure when the analysis was performed for both pre-Neolithic and Neolithic and Neolithic and post-Neolithic, separately. In modern human, BMI, educational attainment in years, fluid intelligence, skin color, skin tanning ease, and standing height show significant levels of selection.

In conclusion, using genetic models applied to ancient genomes, we describe important changes that have taken place in the physiological traits of modern *Homo sapiens* populations living in Europe since the Early Upper Paleolithic. Although the exact extent of the evolutionary changes Europeans underwent remain unclear, based on these genetic trajectories we can see that while the first 40,000 years in Europe represented a period of relative stagnation in the evolution of these traits, the spread of Neolithic populations and of the cultural complex associated with agriculture and animal domestication has led to important changes in height, weight, skin color, an increase in intellectual attainment, as well as in cholesterol metabolism and the risk for cardiovascular diseases. These new insights put into light the processes that have shaped human physiology since the migration of modern humans throughout Europe.

In this study we used PRS constructed with ancient DNA samples as an estimate of a sample’s genetic predisposition for a certain trait, we use this as a pseudo-phenotype to model changes in genetic predisposition for these traits over time. We however do not fold use these PRS to further study the step-wise selection process taking place. Additionally, there are several other factors that can impact the PRS distributions. For example, the power of GWAS studies could impact the accuracy of PRS calculation. There is also a limitation when applying PRS analysis to a population which is different from GWAS participant ancestry from the GWAS studies. Similarly, the socio-economic status (especially for cognition related traits) could be a confounding factor which needs caveats in interpreting results. From the technical point, data imputation was applied for handling missing data, which could limit the power of the study. Also, although there are temporal changes in PRS for various traits coinciding with large changes in lifestyle such as the Neolithic revolution, these results should only be taken as evidence for changes in risk allele frequencies and their effect on the genetic susceptibility for their respective phenotypes. The changes in lifestyle and diet seen in European populations in the same era could be an additional possible explanation, but further research would be required to ascertain this.

## Data Availability

The original contributions presented in the study are included in the article/Supplementary Material, further inquiries can be directed to the corresponding authors.
