# Supplementary figures and images for: Evolutionary Trajectories of Complex Traits in European Populations of Modern Humans
Source: Front Genet. 2022 Mar 28;13:833190. doi: 10.3389/fgene.2022.833190 (PMC8995853; doi:10.3389/fgene.2022.833190)

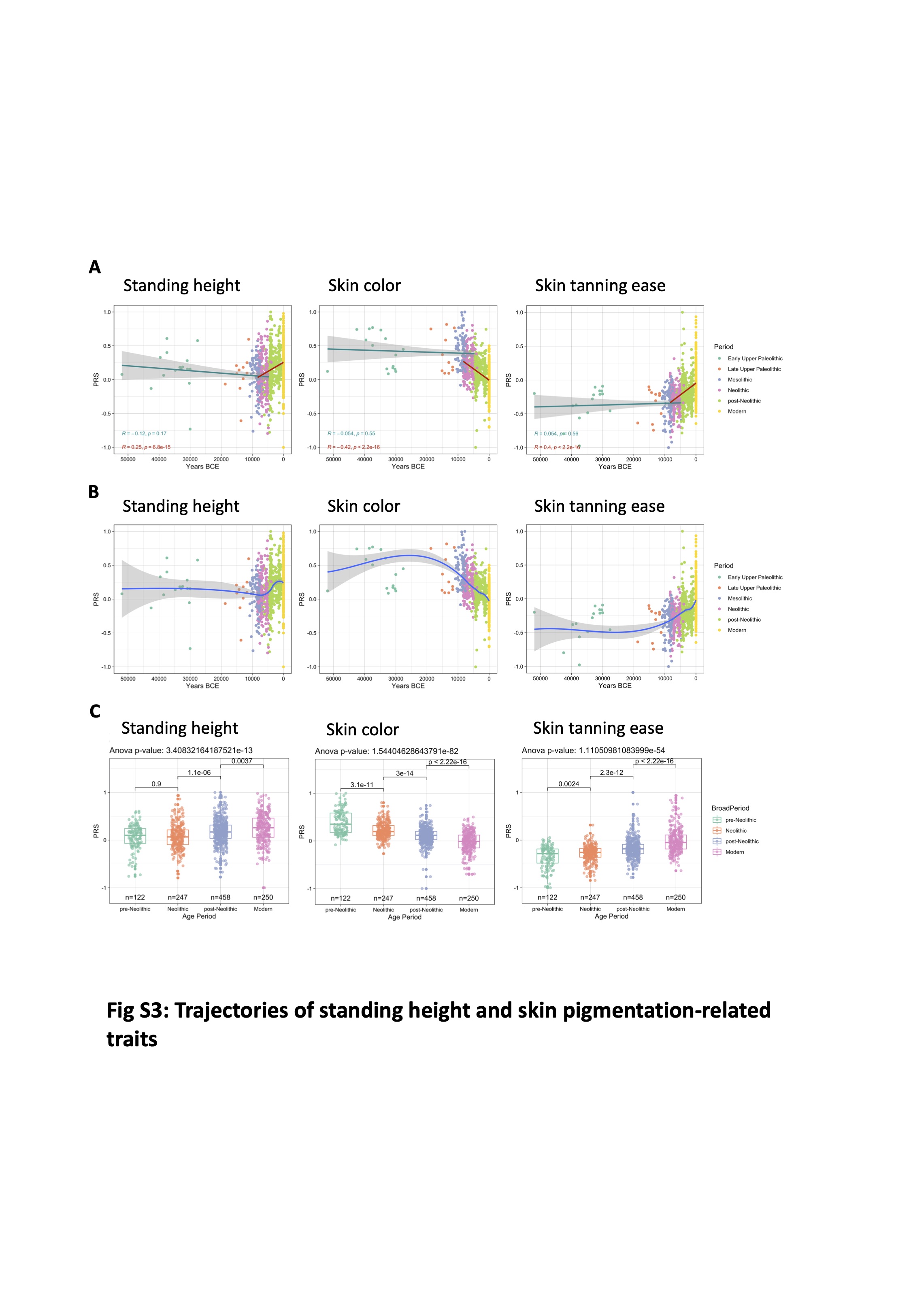

Supplement: Supplementary file 1 [file Image3.JPEG]

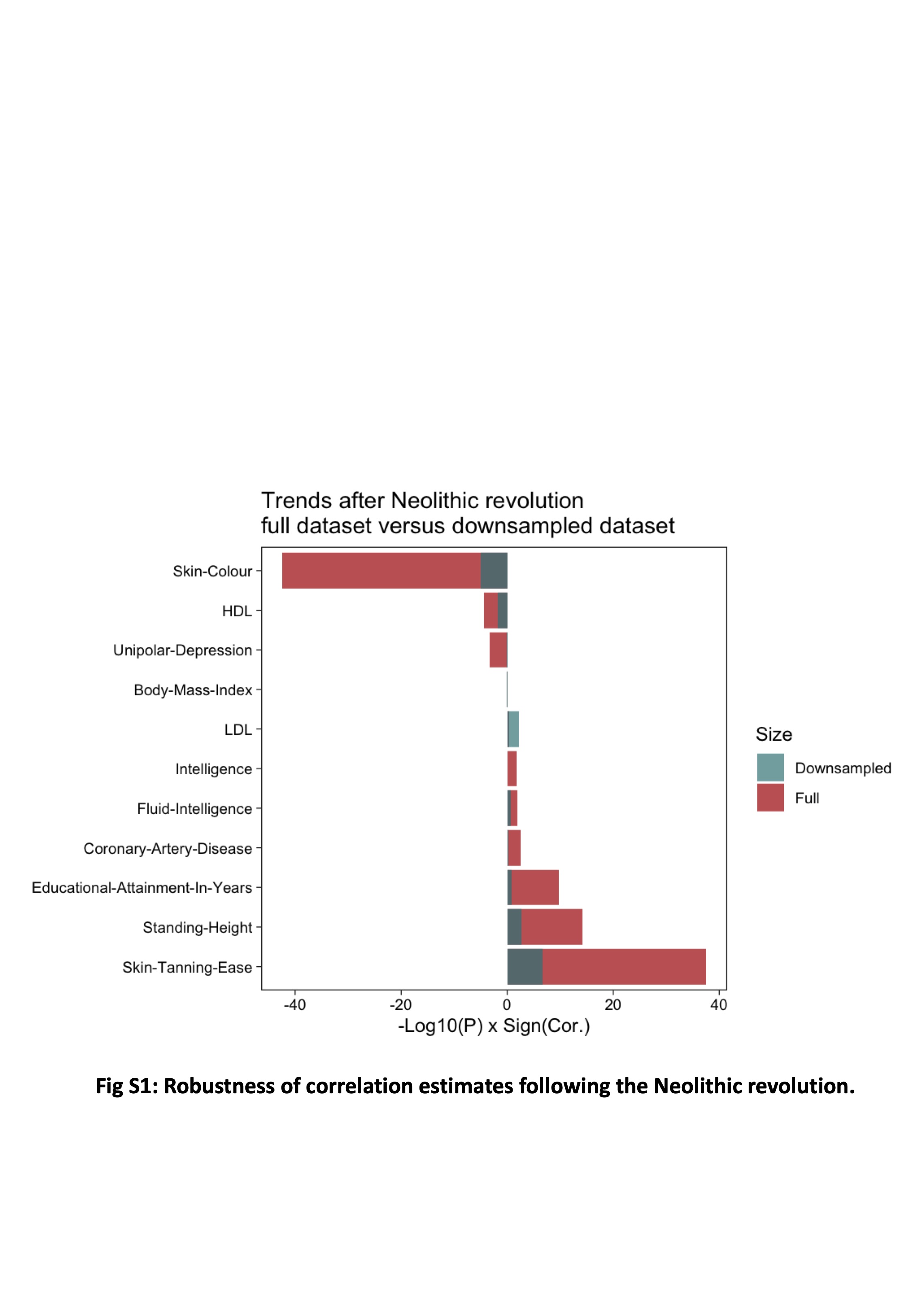

Supplement: Supplementary file 3 [file Image1.JPEG]

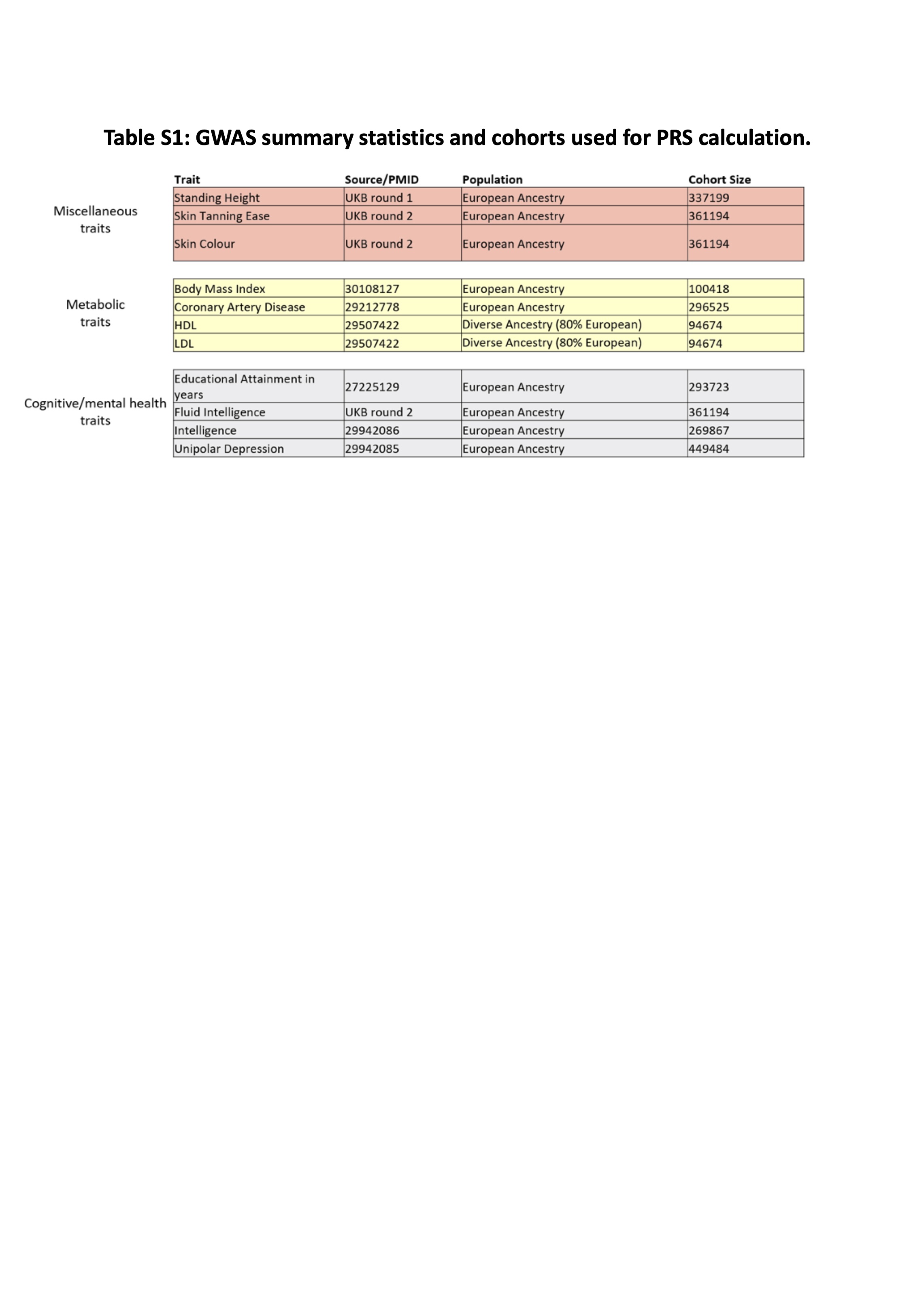

Supplement: Supplementary file 4 [file Image4.JPEG]

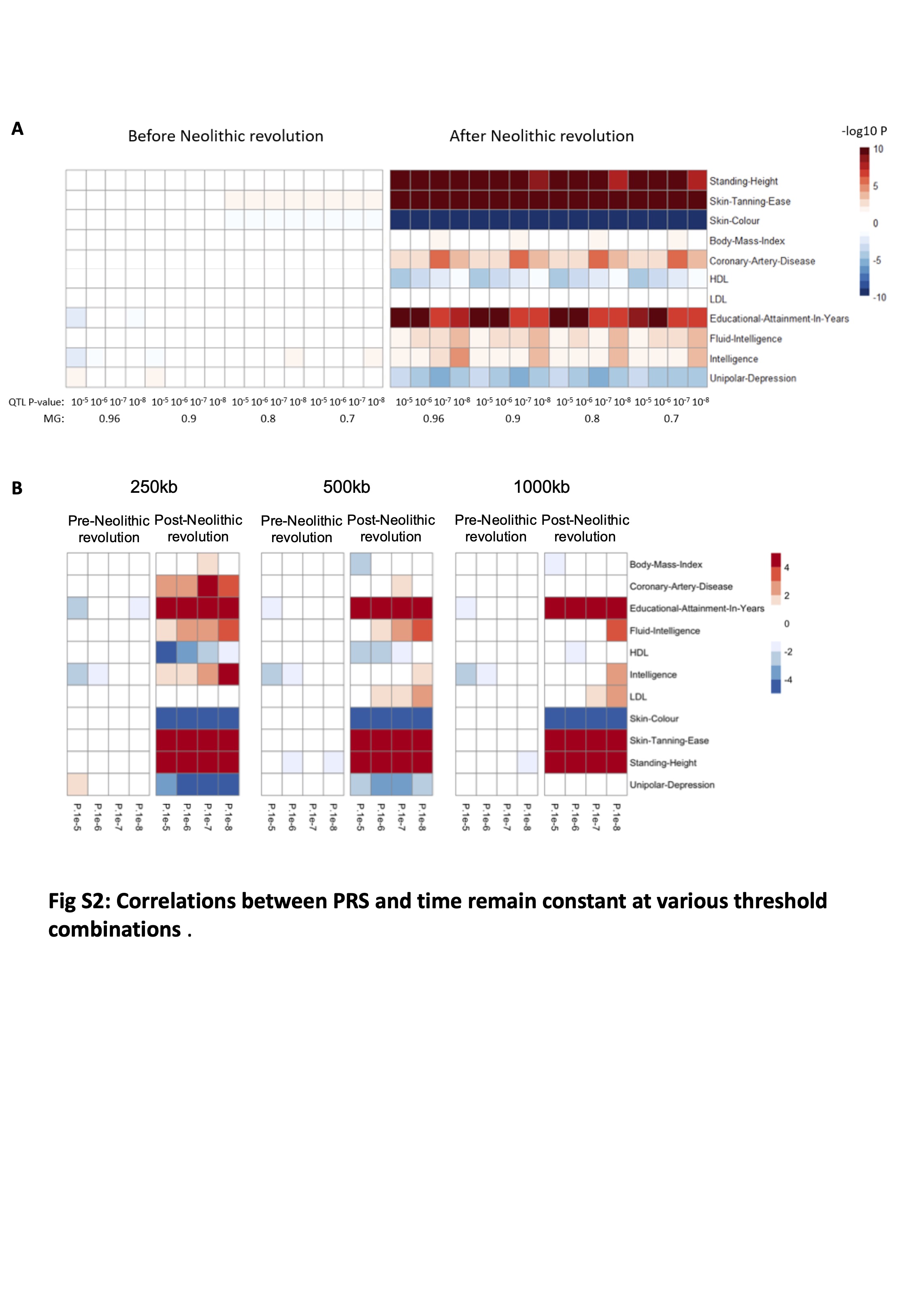

Supplement: Supplementary file 5 [file Image2.JPEG]

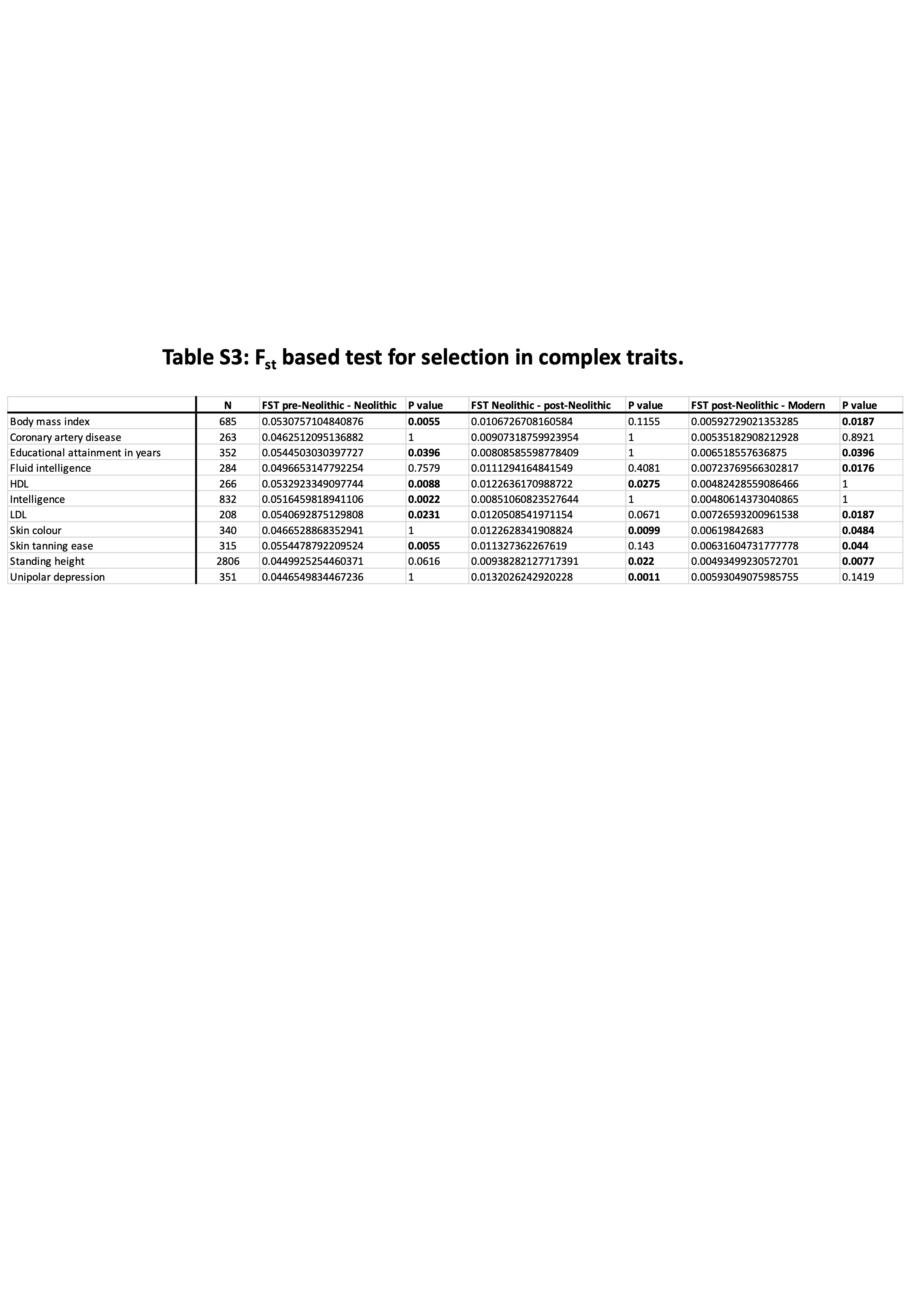

Supplement: Supplementary file 6 [file Image5.JPEG]
