# Supplementary material for: Evolutionary Trajectories of Complex Traits in European Populations of Modern Humans
Source: Front Genet. 2022 Mar 28;13:833190. doi: 10.3389/fgene.2022.833190 (PMC8995853; doi:10.3389/fgene.2022.833190)
Supplement: Supplementary file 2 [file Table1.DOCX]

**Table S2**: Range of heritability of different traits

| **Trait** | **Heritability %** | **References** |
| --- | --- | --- |
| Standing height | 79% | (Geddes, 2019) |
| Skin color | 82% | (Paik et al., 2012) |
| Skin tanning ease | 45% | (Visconti et al., 2018) |
| BMI | 40% | (Geddes, 2019) |
| Coronary artery disease | 40-60% | (McPherson and Tybjaerg-Hansen, 2016) |
| HDL cholesterol | 40-60% | (Weissglas-Volkov and Pajukanta, 2010) |
| Fluid intelligence | 47% | (Davies et al., 2011) |
| Intelligence | 60% | (Plomin and Deary, 2015) |
| Unipolar depression | 42% | (Edvardsen et al., 2009) |
| Educational Attainment | 20% | (Okbay et al., 2016) |

References:

Davies, G., Tenesa, A., Payton, A., Yang, J., Harris, S. E., Liewald, D., et al. (2011). Genome-wide association studies establish that human intelligence is highly heritable and polygenic. *Molecular psychiatry* 16, 996. doi:10.1038/mp.2011.85.

Edvardsen, J., Torgersen, S., Røysamb, E., Lygren, S., Skre, I., Onstad, S., et al. (2009). Unipolar depressive disorders have a common genotype. *Journal of Affective Disorders* 117, 30–41. doi:10.1016/j.jad.2008.12.004.

Geddes, L. (2019). Genetic study homes in on height’s heritability mystery. *Nature* 568, 444–445. doi:10.1038/d41586-019-01157-y.

McPherson, R., and Tybjaerg-Hansen, A. (2016). Genetics of Coronary Artery Disease. *Circulation Research* 118, 564–578. doi:10.1161/CIRCRESAHA.115.306566.

Okbay, A., Beauchamp, J. P., Fontana, M. A., Lee, J. J., Pers, T. H., Rietveld, C. A., et al. (2016). Genome-wide association study identifies 74 loci associated with educational attainment. *Nature* 533, 539–542. doi:10.1038/nature17671.

Paik, S. H., Kim, H.-J., Son, H.-Y., Lee, S., Im, S.-W., Ju, Y. S., et al. (2012). Gene mapping study for constitutive skin color in an isolated Mongolian population. *Exp Mol Med* 44, 241–249. doi:10.3858/emm.2012.44.3.020.

Plomin, R., and Deary, I. J. (2015). Genetics and intelligence differences: five special findings. *Molecular Psychiatry* 20, 98. doi:10.1038/mp.2014.105.

Visconti, A., Duffy, D. L., Liu, F., Zhu, G., Wu, W., Chen, Y., et al. (2018). Genome-wide association study in 176,678 Europeans reveals genetic loci for tanning response to sun exposure. *Nat Commun* 9, 1684. doi:10.1038/s41467-018-04086-y.

Weissglas-Volkov, D., and Pajukanta, P. (2010). Genetic causes of high and low serum HDL-cholesterol. *Journal of Lipid Research* 51, 2032. doi:10.1194/jlr.R004739.
